# A simple, realistic walled phantom for intravascular and intracardiac applications

**DOI:** 10.1007/s11548-020-02201-3

**Published:** 2020-06-10

**Authors:** Hareem Nisar, John Moore, Roberta Piazza, Efthymios Maneas, Elvis C. S. Chen, Terry M. Peters

**Affiliations:** 1 Robarts Research Institute, Western University, London, Canada; 2 School of Biomedical Engineering, Western University, London, Canada; 3 Department of Information Engineering, University of Pisa, Pisa, Italy; 4 Wellcome/EPSRC Centre for Interventional and Surgical Sciences, University College London, London, UK; 5 Department of Medical Physics and Bioengineering, University College London, London, UK; 6 Department of Medical Biophysics, Western University, London, Canada; 7 Department of Medical Imaging, Western University, London, Canada

**Keywords:** Vascular phantom, Walled phantom, Polyvinyl alcohol cryogel (PVA-c), Intravascular imaging (IVUS), Intracardiac imaging (ICE)

## Abstract

**Purpose:**

This work aims to develop a simple, anatomically and haptically realistic vascular phantom, compatible with intravascular and intracardiac ultrasound. The low-cost, dual-layered phantom bridges the gap between traditional wall-only and wall-less phantoms by showing both the vessel wall and surrounding tissue in ultrasound imaging. This phantom can better assist clinical tool training, testing of intravascular devices, blood flow studies, and validation of algorithms for intravascular and intracardiac surgical systems.

**Methods:**

Polyvinyl alcohol cryogel (PVA-c) incorporating a scattering agent was used to obtain vessel and tissue-mimicking materials. Our specific design targeted the inferior vena cava and renal bifurcations which were modelled using CAD software. A custom mould and container were 3D-printed for shaping the desired vessel wall. Three phantoms were prepared by varying both the concentrations of scattering agent as well as the number of freeze–thaw cycles to which the phantom layers were subjected during the manufacturing process. Each phantom was evaluated using ultrasound imaging using the Foresight™ ICE probe. Geometrical validation was provided by comparing CAD design to a CT scan of the phantom.

**Results:**

The desired vascular phantom was constructed using 2.5% and 0.05% scattering agent concentration in the vessel and tissue-mimicking layers, respectively. Imaging of the three phantoms showed that increasing the number of freeze–thaw cycles did not significantly enhance the image contrast. Comparison of the US images with their CT equivalents resulted in an average error of 0.9${\,\mathrm{mm}}$ for the lumen diameter.

**Conclusion:**

The phantom is anatomically realistic when imaged with intracardiac ultrasound and provides a smooth lumen for the ultrasound probe and catheter to manoeuvre. The vascular phantom enables validation of intravascular and intracardiac image guidance systems. The simple construction technique also provides a workflow for designing complex, multi-layered arterial phantoms.

## Introduction

Ultrasound phantoms are widely used in clinical training, pre-procedural planning, academic research methodologies, and industrial device design and testing. Vascular phantoms are widely used in many applications including training on ultrasound-based vascular access [12, 17], study of vascular blood flow dynamics using ultrasound Doppler [4], testing of intravascular catheters [2], as well as the design of image-guided systems for intravascular and cardiac interventional procedures [1, 24].

A wide range of vascular phantoms is described in the literature, from simple, tubular, wall-only vessels [9, 14] to complex, wall-less vascular phantoms surrounded by realistic tissue-mimicking material (TMM). Walled phantoms are easy to fabricate, often using solid rubber-like materials, such as silicone, latex, and C-flex tubing, as vessel-mimicking materials (VMM). Walled phantoms usually have a rough lumen surface, high ultrasound attenuation coefficient, and no TMM in the surroundings, causing them to have an unrealistic appearance in ultrasound and poor haptic response when interacting with an intralumenal device. On the other hand, wall-less phantoms are often fabricated by creating an absence of vessel wall and lumen in a block of TMM. A hollow vessel is created by placing a rigid lumen core and pulling it out once the TMM is set [17], a method that is suitable for simple to moderately complex vessel designs. Highly realistic and intricate vessel geometries can be achieved by constructing the vascular tree from a low-melting point material, surrounding it by a (higher melting point) TMM, and subsequently melting and removing the inner lumen material [15] along with elaborate, resource-intensive, and expensive procedures. Moreover, wall-less phantoms do not incorporate a layer to mimic the vessel wall and hence lack realism when imaged by intravascular (IVUS) and intracardiac (ICE) ultrasound.

Gel-based materials are popular choice of TMMs for wall-less phantoms. Agar [17–19]- and gelatin [20]-based gels have been employed due to their ready availability, but they lack the mechanical durability to maintain the integrity of complex structures. Polyvinyl alcohol cryogel (PVA-c) is another potential option, offering high strength, flexibility, and endurance of external pressures [22]. PVA-cryogel is a water-insoluble hydrogel prepared by mixing water-soluble PVA powder in distilled water over a controlled temperature. Mechanical and acoustic properties of PVA-c can be customized by controlling the number of freeze–thaw cycles (FTC) used in its preparation. A drawback to using PVA-c as a TMM is its sensitivity to heat. Melting the inner lumen material to create a hollow vessel becomes difficult as the heat may also affect the acoustic properties of PVA-c. A pull-out method is therefore preferred to create a hollow lumen when using PVA-c as a TMM.

The ideal characteristics of a phantom are dictated by its intended application. Low-cost phantoms are available in the literature for certain applications, including clinical training of ultrasound needle guidance and procedural training [11, 16, 17]. However, only a few phantom designs are also acceptable for intravascular or cardiac interventional research applications where both vessel and tissue-mimicking layers are required. An ideal phantom for such applications must also be hollow and haptically realistic to allow smooth flow of blood-mimicking fluid (BMF) and proper manoeuvring of tools, catheters, and ultrasound probes. Ultrasound imaging of such a phantom should show the vessel wall distinctly, with liquid flowing inside and the TMM in the surroundings.

**Fig. 1:**
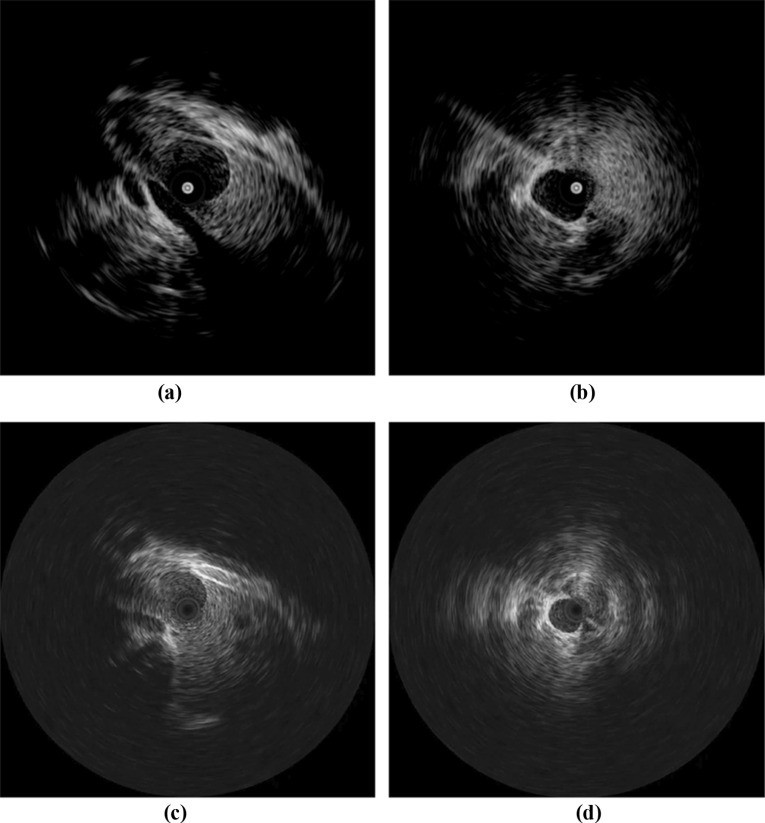
Conavi Foresight™ ultrasound imaging (ICE) of swine inferior vena cava (IVC) showing variations in the appearance of a vessel. Images (**b**) and (**d**) represents the targeted ultrasound imaging aimed in this study. The central dark/bright spot represent the inherent imaging probe artefact

Human and animal vascular and surrounding anatomy is highly complex, and it is often difficult to combine all aspects in one phantom. Ultrasound images of any vessel, tissue, or organ can vary significantly among different subjects and even within the same subject. Vessel wall structures, consisting of tunica intima, media, and externa, have varying proportions of elastic tissue, smooth muscle fibres, and collagen fibrous tissue. The same vessel may have a different appearance under ultrasound as it passes through different regions. For example, the inferior vena cava (IVC) is a long vessel that travels through different anatomical regions of the body. Some parts of this vein located in the abdominal region may appear weakly reflective due to a small proportion of fibrous tissue in tunica externa, while other parts of the vessel that pass through the thoracic region may appear bright under ultrasound. Figure 1 shows some of the variations in the ultrasound imaging of the IVC. Vascular phantom design, like any organ phantom, needs to be specific to the targeted region in the body. In this study, we focused on targeted image in Fig. 1b, d, likely to be acquired when a vessel is ensheathed by a fibrous membrane or surrounded by fatty tissues. Hence, all the design parameters are tailored towards such ultrasound appearance.

This study investigated the use of PVA-c containing a scattering agent to create a two-layered walled vascular phantom combining both VMM and TMM. We experimented with multiple FTCs as well as varying concentrations of scattering agent to obtain the desired ultrasound appearance (Fig. 1d) of the vascular phantom. The overall goal is to develop a simple, low-cost vascular phantom with both a vessel-mimicking layer and surrounding tissue-mimicking material, to obtain anatomically realistic ultrasound imaging, especially under IVUS and ICE.

## Materials and methods

**Fig. 2:**
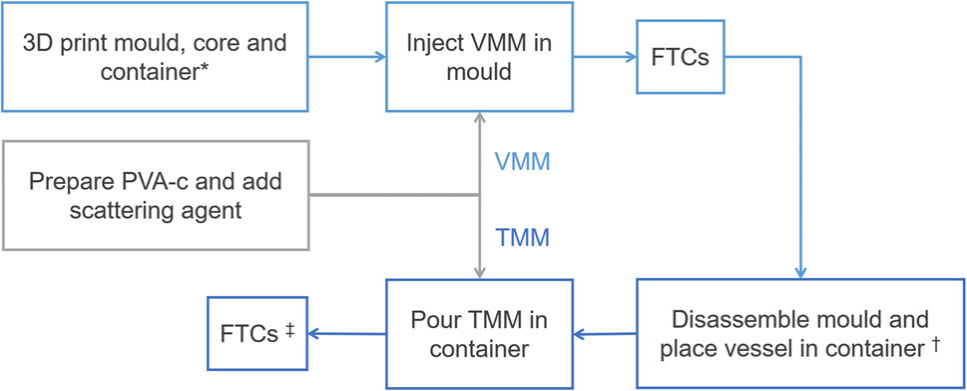
Overall workflow for fabrication of two-layered vascular phantom. Stage 1 involved preparation of vessel-mimicking material (VMM) by mixing polyvinyl alcohol cryogel (PVA-c) with talcum powder as scattering agent and subjecting to freeze–thaw cycles (FTCs). Stage 2 involved preparing and combining tissue-mimicking material (TMM) with solidified VMM. *Figures 5, 6 and 7

The fabrication process for the vascular phantom involved two main stages: the construction of both vessel-mimicking and tissue-mimicking layers. Figure 2 shows the various steps involved in these stages. A positive model of the required vessel wall was used to generate custom mould and container designs, which were 3D-printed in polylactic acid (PLA) thermoplastic. PVA-c was used as a base medium for both vessel and TMMs. Commonly available talcum powder was used as a scattering agent to introduce speckle and backscatter in the vessel and background layer [23]. A solid vessel-mimicking layer was prepared prior to adding the tissue-mimicking material.

Three successive vascular phantoms, A, B, and C, were built sequentially in an iterative fashion, with various parameters for each being revised based on the outcomes of the previous phantom. The aim was to develop a phantom with ultrasound images resembling those obtained from the target anatomy (Fig. 1d). At each iteration, the concentrations of the scattering agent were chosen based on the ultrasound images of the previous phantom. Initial estimates for these concentrations were made based on the expertise of the authors as well as an ongoing study in the laboratory regarding multiple scattering agents in PVA-c. Furthermore, the image contrast between the VMM and TMM layers was investigated by exploiting the change in acoustic impedance that followed the increased number of FTCs. In general, the acoustic impedance of PVA-c increases with the number of FTCs undergone during manufacture. Two PVA-c layers subject to different numbers of FTCs showed distinct appearances in ultrasound. Table 1 summarizes the differences in the three phantoms. Phantom A was made with PVA-c only, with a total of 4 and 2 FTCs for the VMM (vessel wall) and TMM, respectively. Phantom B introduced scattering agent in the TMM and employed a total of 6 and 2 FTCs for the VMM (vessel wall) and TMM. The final version (phantom C) involved generating ultrasound image contrast based on different concentrations (2.5% and 0.05% w/w) of scattering agent in both the vessel and tissue-mimicking layers. Note that, after pouring the PVA-c material for the TMM and subjecting it to a number of FTCs, the VMM is also subject to these additional FTCs.

**Table 1:** Overview of the three phantom versions and their variable parameters scattering agent concentration in VMM and TMM, and number of FTCs vessel-mimicking layer is subjected to before adding TMM

Phantom	Scattering agent concentration		Difference in FTCs between VMM and TMM
	VMM (%)	TMM (%)	
A	0	0	2
B	0	0.1	4
C	2.5	0.05	1

### Mould and container design

Transfemoral access is routinely employed to access targets during cardiac interventions. Surgical catheters and in some cases an ultrasound probe (e.g. ICE) are inserted into the femoral vein, passed through the inferior vena cava (IVC) finally entering the right atrium of the heart. For the purpose of this study, we aim to replicate the geometry of the post-renal portion of the IVC as well as the renal bifurcations. Veins are thin-walled compared to the arteries and can be difficult to mould. Typical measurements for wall thickness of vena cava, veins, aorta, and medium arteries are 1.5 mm, 0.5 mm, 2 mm and 1 mm, respectively [3]. However, these anatomical dimensions of vessels are highly variable between subjects. The vena cava has the largest diameter lumen of any human vein, with an average diameter of 30 mm [3]. The IVC runs from the lower abdominal region to the right atrium in the heart, collecting de-oxygenated blood from multiple organs through tributaries. Along the length of IVC, the lumen diameter, wall thickness, and its appearance in ultrasound can vary significantly depending on the surrounding organs and tissue, with the mean IVC diameter in the infra-renal region being around 20.3 mm [8]. Renal veins have an average diameter of $12\pm$2 mm [21]. Renal veins are not usually orthogonal to the IVC, but instead have a wide range of infra-renal angles, with the IVC, i.e. 15$^{\circ }$–85$^{\circ }$ on the right side and 50$^{\circ }$–90$^{\circ }$ on the left side, with an average of 45$^{\circ }$ and 78$^{\circ }$ with right and left infra-renal angle [10].

**Fig. 3:**
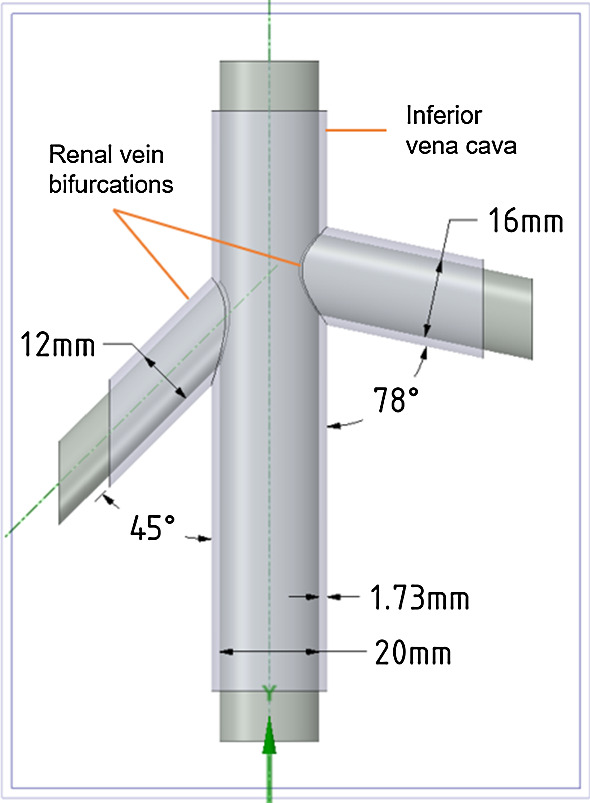
CAD model of vessels representing inferior vena cava (IVC) and renal veins. Extended core for support can be seen at the ends

Vessel structures were modelled in SpaceClaim CAD software (2019 R3, ANSYS, Concord, USA) (Fig. 3). A straight vessel, with an inner diameter of 20 mm, representing the IVC extended 100 mm below the bifurcations. The left renal vein was 33 mm long with a 12 mm inner diameter and placed at an angle of 45$^{\circ }$ with the IVC, while the right renal vein was 37 mm long, with an inner diameter of 16 mm and with infra-renal angle of 78$^{\circ }$. The left renal vein sits 20 mm higher than the right. The targeted wall thickness for the phantom was 1.5 mm. However, to compensate for the shrinking of PVA during FTCs and the slightly oversized prints (sub-millimetre inaccuracy) produced by the 3D printer used in the process, the wall thickness in the CAD model was set to be 1.73 mm.

**Fig. 4:**
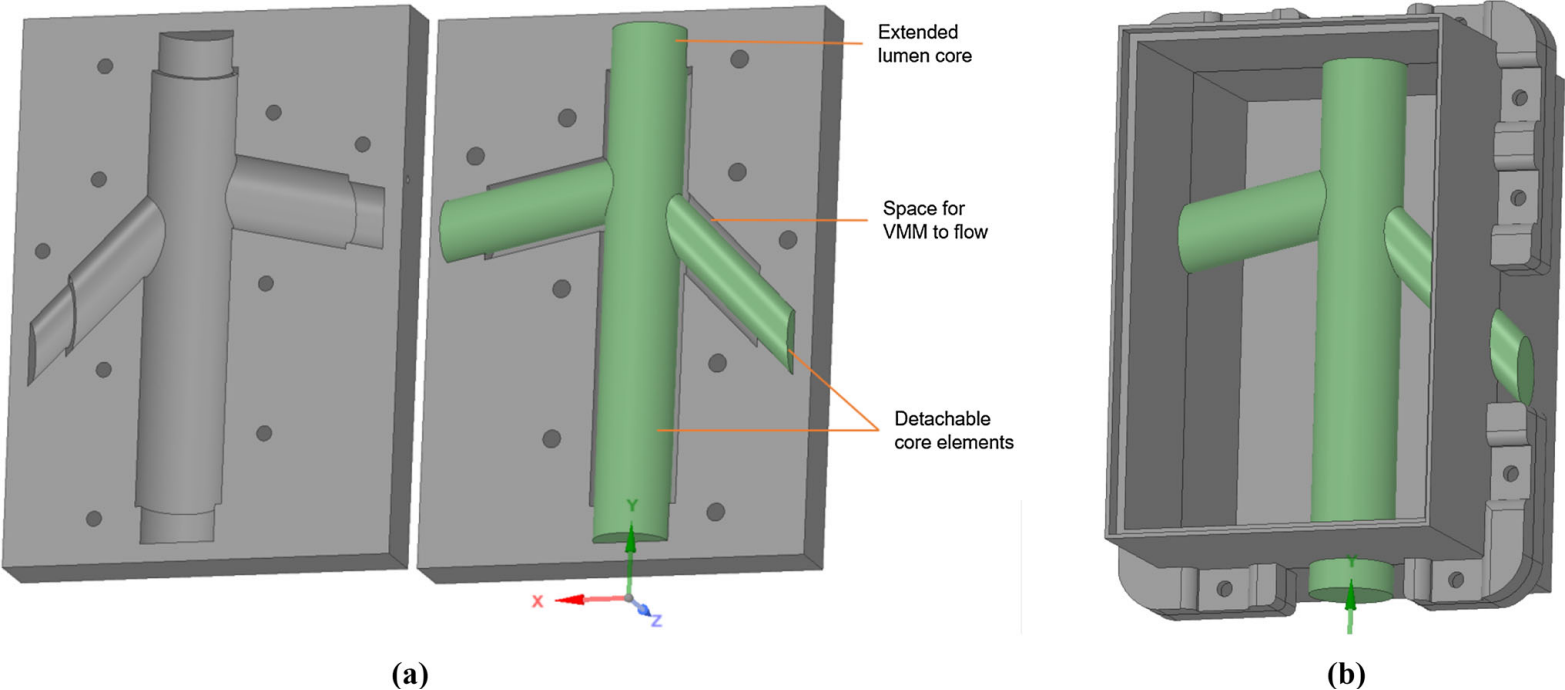
CAD model of **a** mould to be filled with vessel-mimicking material and core elements; **b** container to create a tissue-mimicking block

**Fig. 5:**
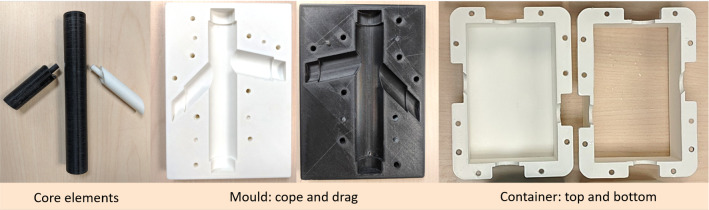
Solid parts of phantom, 3D-printed in poly-lactic acid (PLA) plastic material. From left to right: disassembled core elements with collinear cylindrical joints; cope and drag for the mould; bottom and top half of the custom container

The vessel lumen was elongated to generate support structures for better handling. This inner core design was split into three core elements, with a collinear cylindrical joint, so they could be individually pulled out following the setting of the TMM (Fig. 5). A clam-shell mould was then designed by taking the negative of the core and vessel wall structures, followed by horizontally splitting the negative into cope (top half) and drag (bottom half) structures (Fig. 4). Screws were used to ensure tight closure of the mould. A sprue was made in the cope to allow insertion of fluid VMM through a syringe. Air vents with 2 mm diameter were also made at the top of the vessel and the end of the bifurcations to allow trapped air bubbles to escape from the mould as the VMM is inserted. A custom-made rectangular container (Fig. 4b) was designed for housing the block of tissue-mimicking layer, with separable top and bottom halves, and a space to hold the vessel core elements in the middle of the block.

Mould, core elements, and container were printed in low-cost PLA material using an Ultimaker S3 (Ultimaker, Geldermalsen, The Netherlands). The moulds cope and drag were printed horizontally with the hollow lumen side facing up in order to achieve a smooth cylindrical surface without any support material attachments. Core insert elements were printed vertically for the same reason. 3D-printed parts are shown in Fig. 5. After the printing, screw holes were tapped and a Luer lock was attached to the sprue to create an attachment point for the syringe. Core elements and the inner surface of the mould were gently smoothed by a fine sandpaper to achieve a smooth, curved surface and to remove any irregularities.

### PVA-c preparation

PVA-c was prepared using 10% w/w PVA resin [13]. PVA crystals (Sigma-Aldrich, molecular weight 146000–186000, 99% + hydrolyzed) were mixed with distilled water. For one of the batches, the desired quantity of talc was thoroughly mixed with the water in the conical flask instead of mixing it with PVA prior to freezing, in order to create a more homogeneous mixture. The solution was stirred with an electronic stirrer (Fisher Scientific, Pittsburgh, USA) in a heating mantle (Glas Col, Terre Haute, USA). PVA-c was then set to cool at room temperature before use. Five grams of diazolidinyl urea (>95%) was added to increase the longevity and shelf life of the PVA-c, but this step is not crucial to the construction of phantom and can be omitted.

### Vessel-mimicking layer

**Fig. 6:**
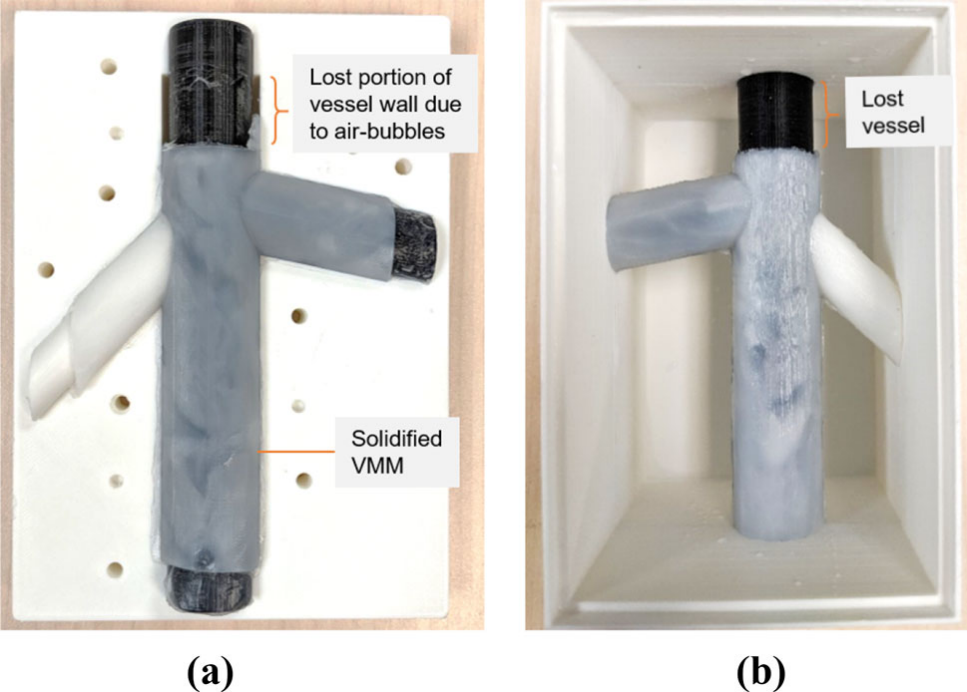
**a** Vessel-mimicking material (VMM) after FTCs, still present inside mould. **b** Solidified VMM with core placed inside the custom container, before filling with tissue-mimicking material. The insert in the oblique bifurcation was printed with white PLA, the other inserts with black PLA

The VMM was prepared by adding the desired percentage (see Table 1) of talcum powder, to previously prepared 10% w/w PVA-c. A syringe was used to insert the VMM into the mould. To solidify the PVA-c vessel wall, the VMM-filled mould was subjected to initial FTCs in an environment chamber (TestEquity model 1007, Moorpark, USA). Each cycle involved instant freezing at – 20 $^{\circ }\hbox {C}$ for 6 h and slowly thawing to 15$^{\circ }\hbox {C}$ for 10 h under controlled conditions using an environment chamber. The mould was disassembled to obtain the solid vessel wall, but the core elements were kept intact at this stage. Figure 6a shows solidified vessel-mimicking layer after two FTCs.

### Tissue-mimicking layer

**Fig. 7:**
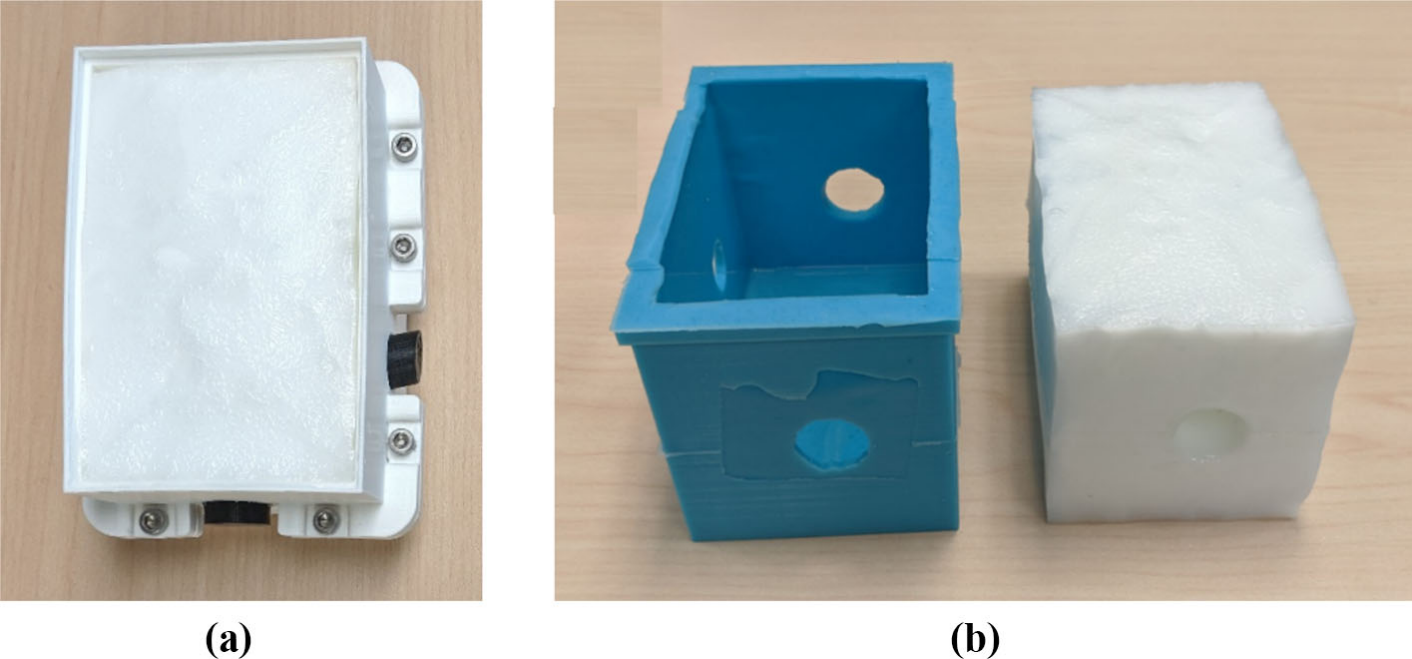
**a** Tissue-mimicking layer after freeze–thaw cycles, still present inside container. **b** Vascular phantom, with tissue and vessel-mimicking layers, accompanied by an optional silicone padding layer

The solidified vessel wall including core elements was correctly positioned and sandwiched between the walls of the 3D-printed plastic container (Fig. 6b). The assembly was held together with metal screws. PVA-c was mixed with desired ratio of talcum powder (see Table 1) to introduce acoustic backscattering and form the TMM. The TMM was poured into the container, covered with plastic wrap to avoid water sublimation due to direct air exposure, and subjected to two FTCs. Each cycle involved freezing at ${-20}^{\circ }\hbox {C}$ for 10 h and slowly thawing to ${15}^{\circ }\hbox {C}$ for 12 h. During the first cycle, the TMM fully adhered to the vessel-mimicking layer as it was solidifying. This attachment was expected as the direct result of cross-linking of PVA polymer chains [5, 25]. Once the phantom construction was complete (see Fig. 7), the core elements were readily extracted and no release agent was required.

### Ultrasound imaging

The phantom was fully submerged in a water bath and scanned with the Conavi Foresight™ ICE intracardiac ultrasound probe (Conavi Medical Inc, Toronto, Canada) [6]. Foresight™ is a forward-looking, radial ICE probe that uses a single-element mechanically rotating—transducer to produce B-mode, conical surface images. The probe was inserted inside the vessel lumen and moved along its length. Images were acquired at 12MHz, and a 55 $^{\circ }$–70 $^{\circ }$ tilt angle, and an imaging depth of 5 cm and 8 cm radially. The phantom was padded with a silicone boundary as sound dampening material to remove any ringing artefact that could arise from the PVA-c to water boundary. Alternatively, time gain compensation (TGC) could be applied at the distal end of the radial image to suppress the signal and remove the appearance of phantom edges in the image. This adjustment was not essential for imaging the phantom, but it served to render the images in a more realistic manner and enable the observer to focus on the vessel of interest.

The ICE images obtained from the phantom were compared with those obtained from porcine experiments. Imaging experiments on Yorkshire swine, approximately 40 kg in weight, were performed under a protocol approved by Sunnybrook Research Institutes Animal Care Committee

### Computed tomography (CT) imaging

A CT scan of the PVA-c phantoms was performed to validate the phantom design technique by quantifying their geometrical properties. O-arm (Medtronic, Dublin, Ireland) standard settings for the HD scan for a small head protocol (with 100 kVp, 20 mA, and 250 mAs) were used. Reconstruction was performed on a Medtronic mobile station using their proprietary software. The voxel resolution was 0.415 $\times$ 0.415 $\times$ 0.833 mm. The lumen diameters for the three vessels mimicking IVC, left renal vein and right renal vein were measured, as well as the infra-renal angles for the bifurcations.

## Results

**Fig. 8:**
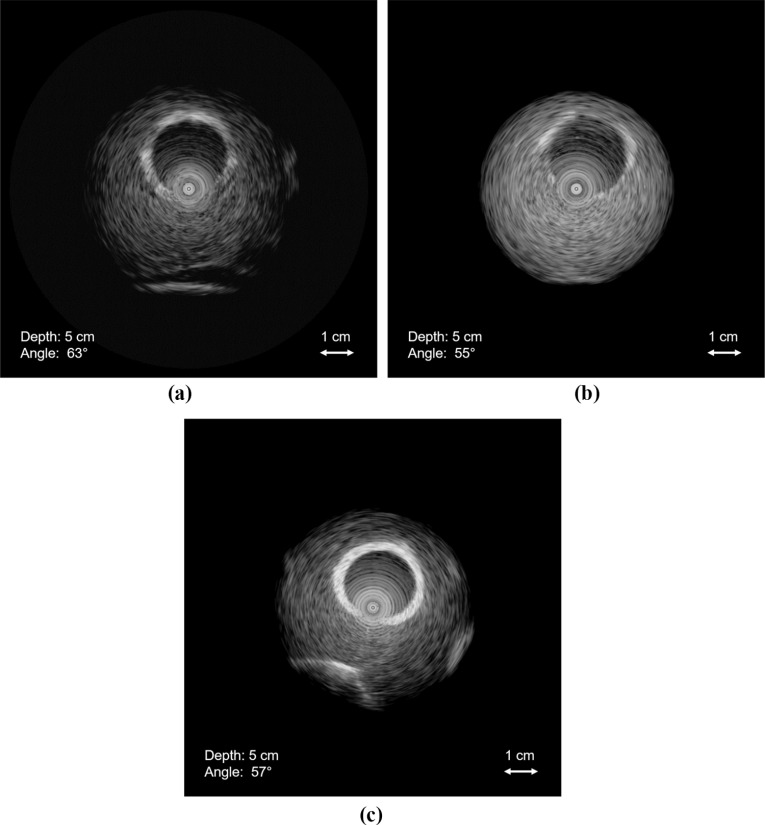
Conavi Foresight™ intracardiac ultrasound (ICE) imaging of **a** phantom A, **b** phantom B and **c** phantom C, at a radial depth of 5 cm and with time gain compensation (TGC) to suppress the edges of phantom. Phantoms A and B show weak reflections from the vessel-mimicking layer, while B depicts increased backscatter from tissue-mimicking material. Phantom C shows strong reflections from the vessel-mimicking layer. Concentric circles in the middle represent inherent imaging probe artefact

The designed vascular phantoms exhibited mechanical strength and flexibility and were able to endure external pressures and retain their shape after minor bending. In terms of haptics, the phantoms were slippery to the touch when placed in water, while the ultrasound probe and catheters were easily manoeuvred inside the lumen. Conavi Foresight™ ICE probes were used to image these three phantoms. Imaging of phantom A (i.e. without scattering agent) depicted weak contrast between the vessel and tissue-mimicking layers. The vessel walls did not show up brightly, and there was minimal backscatter in the tissue-mimicking layer. Vessel bifurcations and the water-filled lumen could be seen clearly in the image. Figure 8a shows the imaging of phantom A at a tilt angle of 56$^{\circ }$, and radial depth of 5 cm, along with TGC to suppress the phantom edges. Note that the circular patterns in the middle of the image represent an artefact inherent to the ultrasound probe and should be ignored.

Phantom B (i.e. with a difference of four FTCs between the VMM and TMM, and the incorporation of scattering agent in TMM) was initially expected to have a sharper, brighter looking vessel wall because of the increased number of freeze–thaw cycles, but imaging showed otherwise. Figure 8b shows a weak contrast between the two layers of the phantom, and the tissue-mimicking layer appears somewhat bright and heavily speckled in the ultrasound and appears unrealistic.

**Fig. 9:**
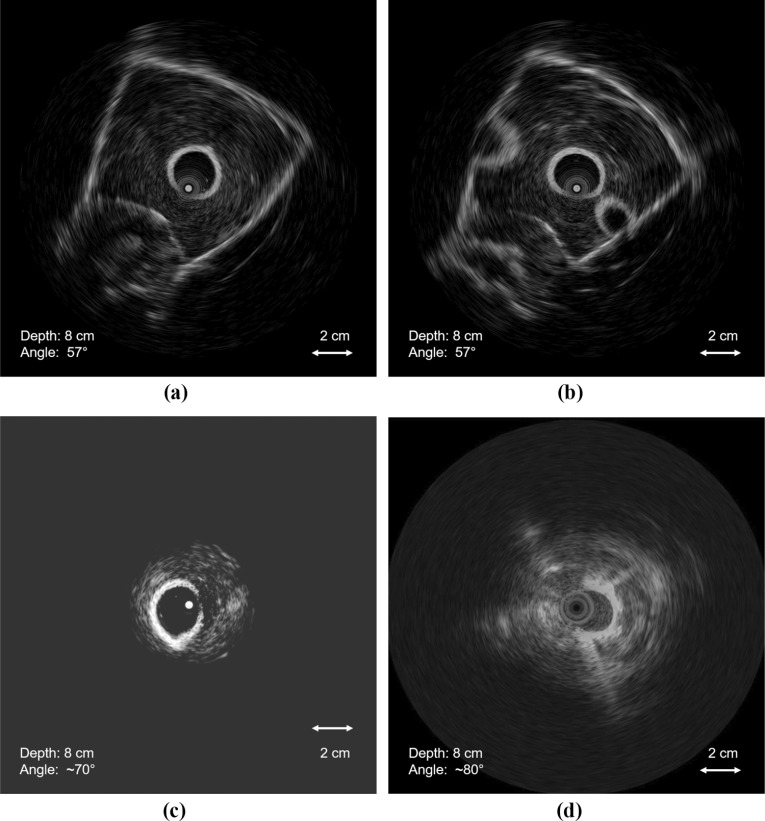
Conavi Foresight™ ultrasound imaging (ICE) of phantom C (**a**, **b**) at a radial depth of 8 cm showing main vessels and bifurcations, (**c**) and with time gain compensation (TGC), showing strong reflections from the vessel-mimicking layer and adequate scattering in the tissue-mimicking material. **d** Swine inferior vena cava (IVC) imaged using Foresight™ ICE at a radial depth of 8 cm

The final phantom (C, with 2.5% and 0.05% scattering agent in VMM and TMM, respectively) produced bright reflections from the vessel wall and adequate speckle in the tissue-mimicking layer (Fig. 8c). Bifurcations could be seen properly as well. Figure 9a, b shows the ultrasound imaging of phantom at the depth of 80 mm without any TGC. Comparison with ICE images of the porcine IVC revealed phantom C to be the most promising design. Figure 9c, d compares our vascular phantom representing IVC and a swine IVC image, each acquired using different Conavi Foresight™ ICE probes. Relative contrast observed in the phantom images is evaluated against the targeted in vivo animal IVC image. Ultrasound image of phantom C (Fig. 9a) showed a tissue to vessel layer pixel intensity ratio of 1 : 1.9, as compared to the ratio of 1 : 1.7 observed in the swine IVC image (Fig. 9d).

**Fig. 10:**
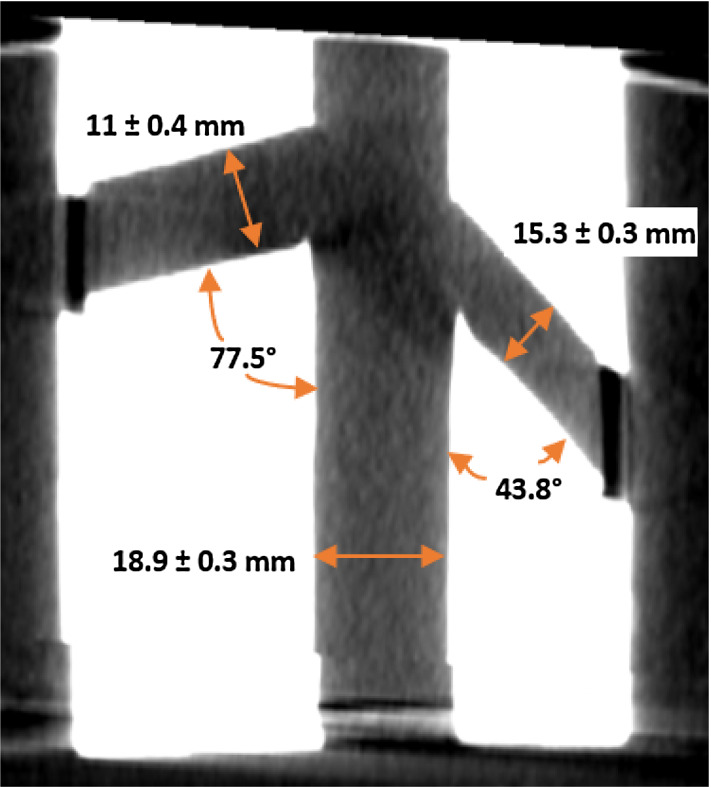
Cross section of the CT scan of the phantom with average lumen diameter of the main vessel and bifurcations, and infra-renal angle measurements

A CT scan of one of the phantoms was used to measure the lumen diameter (see Fig. 10). An average of ten measurements across each vessel revealed a mean error of 1 mm, 0.9 mm, and 0.7 mm for the IVC, left renal vein, and right renal vein, respectively. The left infra-renal angle was measured at $43.8^{\circ }$ compared to the CAD designed value of $45^{\circ }$ and the right infra-renal angle was $77.5^{\circ }$ as compared to the CAD designed $78^{\circ }$.

## Discussion

In this study, the use of PVA-c was investigated to construct two-layered, walled, vascular phantoms. Talcum powder was used as a scattering agent and mixed with PVA-c to form vessels and TMMs. We designed a phantom with the geometry of IVC and renal bifurcations, imaged it using a Conavi Foresight™ intracardiac ultrasound probe and compared it with images obtained from a porcine IVC. We observe the contrast in ultrasound due to (1) varying acoustic properties of PVA-c related to the number of FTCs applied during the solidification process and (2) the brightness achieved by the scattering agent.

In vivo ultrasound imaging of vasculature is highly variable as shown in Fig. 1. The phantom results are a representation of imaging observed in some parts of in vivo imaging of human vasculature. Based on the ultrasound imaging of the three phantoms, recommended values for the varying parameters to acquire ultrasound images, such as in Fig. 1d, are given in Table 2. Note that these values are specific for our designed phantom to obtain the target images. The scattering agent concentrations in the VMM and TMM may vary when aiming for other vessels or some other parts of IVC. The concentrations used in this study, along with the images (Fig. 8a–c), can nevertheless provide a good basis of a starting point to construct other similar vascular structures. This study focuses on a single application, and future work is required to consider an exhaustive range of scattering agent concentrations.

Adding large quantities of talcum powder to prepared PVA-solution can cause minor clumping, and over-mixing introduces more air bubbles into the mixture. It is recommended to add the scattering agent to distilled water during the PVA-solution preparation to achieve a uniform, homogeneous mixture. Regardless of the stage at which talcum powder is added, the PVA-talcum mixture should not be allowed to sit for more than a few hours; otherwise, the talcum will settle at the bottom making the mixture heterogeneous and will require further stirring. Stirring PVA-solution, especially before adding to an intricate vessel wall mould, can potentially introduce air bubbles into the fluid VMM. Figure 6 shows that some portion of the vessel wall was lost due to the collection of air bubbles at the top. It is suggested that extra room in the vessel design be left at the top for air bubbles to rise and occupy this space. This extended portion of the vessel wall can be snipped after solidification of VMM.

The number of FTCs changes the mechanical and acoustic properties of PVA-c. According to [7], mechanical properties of PVA-c linearly vary with the increase in the number of FTCs. However, most curves of FTC-dependent properties achieve a plateau after four freeze–thaw cycles. As seen from phantom B image (Fig. 8b), a large difference in the number of FTCs did not drastically affect the contrast between the two layers. Scattering agents, on the other hand, seems to be more efficient at generating bright reflections and contrast in an ultrasound image. Nonetheless, a minimum of two FTCs are recommended for the inner, vessel-mimicking layer for better structural integrity of the phantom.

One of the limitations of our phantom is the homogeneity of the tissue-mimicking layer. Comparing phantom image (Fig. 9c) with the targeted swine IVC image (Fig. 9d), we observe several differences. The phantom image is plain, homogeneous in both layers, and there is a sharp boundary between the vessel and surrounding tissue, while the animal image has random bright speckles in the surrounding tissue region. The swine IVC image is likely acquired when the vessel is close to a fatty region in the animal body, causing bright speckles in the surrounding tissue as well as brighter reflections from the vessel wall. It must also be kept in mind that the two images are taken using different ultrasound probes (Conavi Foresight™ ICE) and different parameters such as gain, frequency, and time-gain compensation. The centre of all these images appears significantly different because of an absolute bright or dark circle, surrounded by concentric rings. These artefacts, inherent, and unique to each Foresight™ ICE probe, are a result of near-field noise in the ultrasound probe.

The technique was validated using a CT scan. CT imaging of the phantom showed that this is a promising method for reproducing moderately complex vessel geometries. Measurements of vessel lumen diameters from the CT, when compared to the targeted dimensions, report an overall error of 0.9 mm. An error of approximately $1^{\circ }$ is observed between the targeted and measured infra-renal angles. We believe that these discrepancies are introduced by the non-rigid nature of PVA-c, which results in slight deformation of the phantom when compressed due to its weight. Note that the phantom lumen diameters were always less than the targeted values. This behaviour is most likely caused by the fact that PVA-c tends to shrink when exposed to air, and our phantoms were kept in an open environment for an hour before CT imaging. This attribute brings to light a limitation for all PVA-c phantoms that they required to be handled with extreme delicacy. Particular attention must be paid when using and storing PVA-c, to ensure that it is either submerged in water or maintained in a humid air-tight container.

Quantification of acoustic properties of a phantom is highly significant, especially when the phantom is intended to be a direct substitute for a tissue vessel, organ, etc. There is extensive literature available on the quantitative analysis of PVA-c and its use in the construction of phantoms for biomedical applications. A summary can be found in [25]. Acoustic properties of PVA-c have been studied as a function of the number of freeze–thaw cycles. [22] concludes that while the speed of sound in PVA-c is comparable to that in human soft tissue $({1540}\hbox { m.s}^{-1})$, the range of attenuation coefficient ($0.075-{0.28}\,\mathrm{dB\, cm}^{-1}\, \hbox { MHz}^{-1}$) does not correspond to the rule of thumb of ${1}\,\mathrm{dB\, cm}^{-1}\, \hbox { MHz}^{-1}$ for tissue. This means that PVA-c is not ideal to directly substitute for human tissue. Despite this issue, PVA-c has nevertheless been employed effectively for this purpose in numerous applications. The differences in attenuation can easily be compensated for by adjusting different parameters on an ultrasound machine such as frequency, gain, and TGC. Hence, while it would be useful, it is perhaps not absolutely necessary to match all acoustic properties of a phantom designed only for imaging.

The vascular phantoms described here were sturdy and easy to handle. The smooth surface of PVA-c makes it ideal for fabricating a vessel phantom compared to silicone or tube-based vessel phantoms, which have a tacky resistive surface. This characteristic allows for simulating intravascular procedures on a phantom with realistic imaging and haptic characteristics. The layered structure of the phantom presents an opportunity to make more complex, multi-layered arterial phantoms [2], using multiple doping agents or in different concentrations.

## Conclusion

Vascular phantoms have been employed in many different medical applications. Their design, development, and fabrication strategy strongly depend on the final purpose of the phantom. In this paper, we presented a low-cost and repeatable methodology to build a hollow, walled vascular phantom, employing a simple method to obtain a replica of blood vessels, bridging the gap between walled and wall-less phantoms. Using only PVA-c and scattering agent, the ultrasound response of some parts of the IVC was satisfactorily replicated, clearly demonstrating the desired characteristics of a bright vessel wall, vessel bifurcations and weakly reflected TMM in the surroundings.

**Table 2:** Recommended values for talcum powder concentration in 10% w/w polyvinyl alcohol cryogel (PVA-c) to form vessel-mimicking material (VMM) and tissue-mimicking material (TMM), and the number of freeze–thaw cycles (FTCs), the VMM should be subjected to before adding TMM

Recommendations for targeted vascular phantom (representing part of inferior vena cava)		
Talcum powder concentration	TMM	$>2.5\%$
	VMM	$< 0.1\%$
Number of FTCs for vessel wall only		2
